# Insulinoma Presenting as a Complex Partial Seizure: Still a Possible Misleading Factor

**DOI:** 10.3389/fnins.2019.01388

**Published:** 2020-01-17

**Authors:** Ziyou Qi, Daojing Li, Jinfeng Ma, Peng Xu, Yongnan Hao, Aimei Zhang

**Affiliations:** Department of Neurology, Affiliated Hospital of Jining Medical University, Jining Medical University, Jining, China

**Keywords:** insulinoma, complex partial seizure, hypoglycemia, neuroglycopenic, pancreas

## Abstract

Delayed diagnosis of insulinoma remains an intractable clinical challenge because the symptoms are in most cases misattributed to other disorders. In this study, a 64-year-old man presented with intermittent seizure episodes after being misdiagnosed with epilepsy and receiving anti-epileptic drugs for 4 years. During this period, the patient continued to suffer from repeated seizures. A starvation test, pancreatic enhancement CT, MRI scan, and pathological examination clinically diagnosed insulinoma, and the symptoms improved following surgical removal of the tumor. The appearance of unusual manifestations and insulinoma imaging makes it difficult to accurately diagnose the condition. This case emphasizes the need for careful reassessment of all atypical and refractory seizures for neurologists.

## Introduction

Insulinoma is a very rare neuroendocrine tumor with a reported incidence of 0.5–5 per million person-years. It is also the most common cause of hypoglycemia associated with endogenous hyperinsulinemia (Marks, 1971). Clinical clues suggest that insulinoma continues to be diagnosed based on the physician’s recognition of the presence of hypoglycemic symptoms, such as sweating, hunger, tremors, and palpitations. When the relationship between symptoms and possible hypoglycemia is missed, in most clinical settings, the blood glucose levels are not be checked. In addition, hypoglycemic symptoms are varied, lack specificity, and mimic many common neuropsychiatric disorders, such as epilepsy (Graves et al., 2004).

Complex partial seizures are characterized by an aura, impaired consciousness, automatisms, and sometimes psychopathology, also known as temporal lobe seizures (TLE) or psychomotor seizures. Sometimes they are easily confused with metabolic diseases, such as hypoglycemia (Graves et al., 2004).

In this study, we report a case of insulinoma with impaired consciousness and behavioral disorders, which resulted from hypoglycemia and which were misdiagnosed as complex partial seizures based on the normal fasting blood glucose and glycosylated hemoglobin levels prior to admission. In clinical practice, for atypical complex partial seizures, in addition to eliminating epilepsy, the idea should be broadened and positive; finding other causes and thinking of differentiation from extracranial diseases, such as insulinoma, came to our mind.

## Case Presentation

A 64-year-old male patient was referred to our department at the Affiliated Hospital of Jining Medical University for management of refractory seizures. The patient first visited the hospital in 2013 and presented with disturbance of consciousness and behavioral abnormalities with no obvious family or social history. The patient also suffered from palpitations, unclear vision, or dizziness for about 3–5 min, and these were later characterized by impaired consciousness and automatisms. The patient would also remain unresponsive for up to 30–60 min before he would recover spontaneously with no distortion of the commissures. Based on these symptoms, the patient was initially diagnosed with epilepsy at a different hospital and subsequently received regular treatment with oxcarbazepine, an antiepileptic medication. Despite the use of different antiepileptic drugs (AEDs) the patient continued to have 3–5 attacks per year.

**FIGURE 1:**
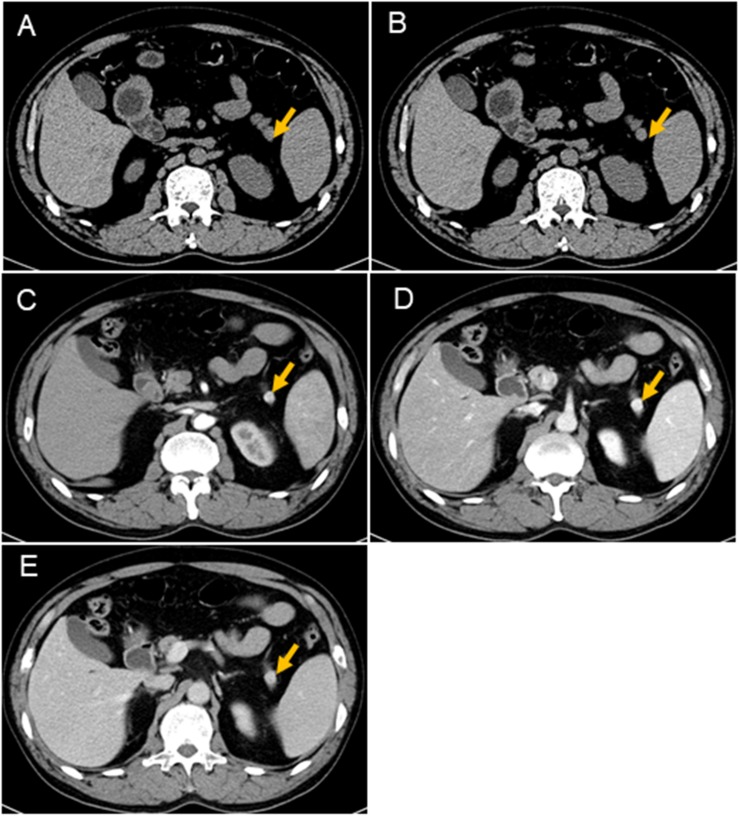
CT scans of the pancreas. Nodules (arrows) can be seen in the tail of the pancreas. It is not on the same level as the pancreas, which results show a lack of the same layer contrast. The CT values of the nodules are similar to the CT values of the pancreas **(A,B)**. The size of the mass is about 1.1 cm × 1.2 cm, and the boundary is clear. The mass is significantly enhanced at the arterial phase, and the CT value is about 125HU **(C)**; the parenchymal phase is significantly enhanced, and the CT value is about 170HU **(D)**; the venous phase is significantly enhanced, and CT value About 164 HU **(E)**.

**FIGURE 2:**
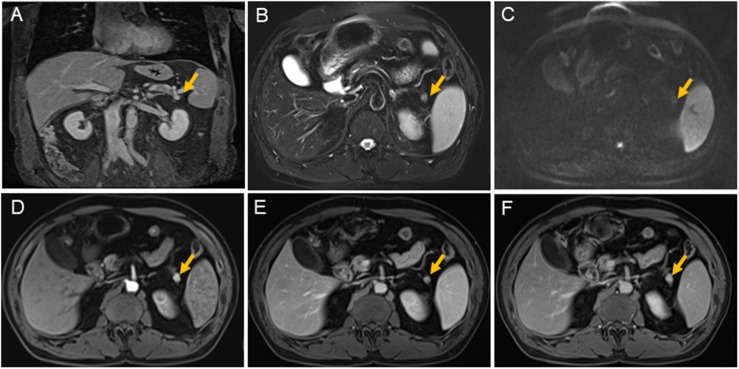
Magnetic resonance imaging scans of the pancreas. There is a nodule about 1.1 cm × 1.2 cm (yellow arrow) with clear margin in pancreatic tail. The nodule is shown isointensity on T1 weighted-image **(A)**; on T2 weighted-image, it is demonstrated hyperintensity **(B)**; it is shown slight restricted on diffusion weighted image **(C)**; it is shown contrast-enhancement on early arterial phase **(D)**; also on balance phase **(E)**; and continuing contrast-enhancement on venous phase **(F)**. The main pancreatic duct is not expanded. The size and form of liver, gallbladder, and spleen is not unusual, the signal is normal.

**FIGURE 3:**
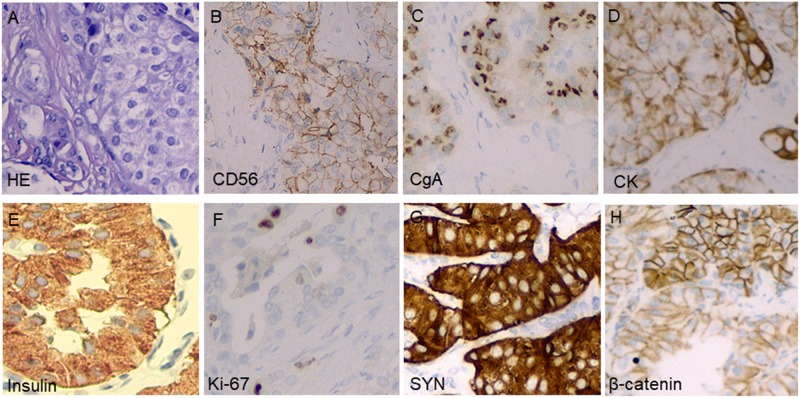
Pathological examinations confirmed the tumor in the pancreatic distal region to be insulinoma. Hematoxylin and eosin stain of a cell-block preparation shows bland cells that are arranged in vaguely nested architecture, a characteristic appearance of neuroendocrine tumors **(A)**; diffusely positive CD56 staining on an immunostaining analysis **(B)**; diffusely positive chromogranin A staining on an immunostaining analysis **(C)**; diffusely positive CK staining on an immunostaining analysis **(D)**; diffusely positive insulin staining on an immunostaining analysis **(E)**; approximately 1–2% of tumor cells stained positive for Ki-67 **(F)**; diffusely positive SYN staining on an immunostaining analysis **(G)**; and diffusely positive β-catenin staining on an immunostaining analysis **(H)**.

At admission, a physical examination, neurological examination, brain magnetic resonance imaging (MRI), and electroencephalogram (EEG) showed no obvious abnormalities. Laboratory test results revealed normal serum fasting glucose levels (5.3 mmol/l), glycosylated hemoglobin levels (5.1%), and ammonia levels (<8.7 umol/l). Sodium potassium chloride, calcium, magnesium, and phosphorus levels showed no obvious abnormalities. Continuous glucose monitoring (CGM) also showed no abnormalities during the first 3 days after admission. However, on the fifth day after admission, the finger prick test revealed a blood glucose level of 2.5 mmol/L before lunch, and this was lower than the normal value despite the patient not having any hypoglycemia-related symptoms, such as palpitations, sweating, and hunger. Based on the Whipple’s triad [consists of episodic hypoglycemia (<50 mg/dL), symptoms of hypoglycemia include confusion, anxiety, paralysis, stupor, coma, and reversal of symptoms with glucose administration], and the seizure-like symptoms, we considered that the possibility of an endocrine disease should be ruled out. A laboratory examination showed that cortisol rhythm (8 am, 4 pm, and 0 am) was 13.37, 2.16, and 10.68 ug/dl, and ACTH rhythm (8 am, 4 pm, and 0 am) was 12.86, 4.97, and 9.10 pmol/L, respectively, and these were all within the normal range. The patient was then examined for insulinoma. Islet cell antibody was weakly positive, anti-glutamate decarboxylase antibody was 0.62 U/ml (0–1 U/ml), and the hunger test was performed by allowing the patient to fast after dinner and have their blood glucose levels monitored every 2 h. Laboratory examination of the blood glucose levels revealed that fasting glucose (6 am) was 2.9 mmol/L, serum insulin (6 am) was 17.47 uIU/ml (reference range 2.6–24.9 uIU/ml); fasting glucose (11:30 am) was 2.0 mmol/L, serum insulin (11:30 am) was 13.50 uIU/ml, and insulin/blood glucose > 0.4. An abdominal CT scan showed a 1.5 cm mass in the tail of the pancreas (Figures 1A,B). However, since the mass was not located at the same level as the pancreas, and the CT value of the lesion (44Hu) was relatively similar to the CT value of the pancreatic parenchyma (35Hu), this led to misdiagnosis of the tumor. Further pancreatic CT showed significant enhancement of the nodular arterial phase with a slight withdrawal from the delayed phase (Figures 1C–E). An MRI-enhanced scan of the upper abdomen showed a slightly higher signal of T1WI in the tail of the pancreas (Figure 2A) and a high signal of T2WI (Figure 2B) with a diameter of about 1.2 cm and showing mild progressive enhancement (Figures 2C–F). The CT and MRI examination suggested that islet cell tumor might occur. The size and form of liver, gallbladder and spleen is normal. The patient was subsequently transferred to the department of hepatobiliary surgery to undergo surgical removal of the tumor. A histological analysis confirmed the excision of a benign pancreatic insulinoma with a Ki-67 labeling index of 1–2%, which indicated a low risk of malignant behavior. The tumor was positive for CK+(CKLow), CgA(+), CD56(+), Syn(+), insulin(+), and β-catenin(+) (Figure 3). CK is the main tag of the simple and glandular epithelium, SYN, CgA, and CD56 are used to identify tumors arising from neural and neuroendocrine tissues, and positivity for insulin provides tumor-specific confirmation of the disease. Following surgical removal of the tumor, the patient’s blood glucose level normalized, and no recurrence of seizures was noted.

## Discussion

In this study, we reported a case of insulinoma presenting as a refractory seizure disorder in adulthood. The patient experienced the first attack 4 years ago. The atypical features of the attacks were inconsistent with complex partial seizures and poor response to treatment, which prompted an inpatient assessment. This case highlights the importance of considering hypoglycemia in atypical and refractory seizures.

Hypoglycemia is a well-recognized cause of acute symptomatic seizures. Several cases of patients with recurrent seizures due to insulinoma-associated hypoglycemia have been reported (Mohseni, 2001).

Insulinomas are the most common hormone-secreting tumors of the gastrointestinal tract and were first discovered by Nicholis in autopsy in 1902. The incidence of 0.5–1 cases/million/year. The diagnostic criteria rely on inappropriate insulin secretion (0.30 pmol/l), which is consistent with hypoglycemia (2.2 mmol/l) and subsequent tumor localization (Okabayashi et al., 2013). Islet cell tumors can be categorized as functional islet cell tumors and non-functional islet cell tumors based on the presence or absence of endocrine function. Non-functional islet tumors account for about 65% of the islet cell tumors; however, they lack specificity in clinical manifestations, and metastasis has already occurred in most diagnoses. Functional islet cell tumors are rare and can be divided into insulinoma, gastrinoma, glucagonoma, vaso-intestinal peptide tumors, and so on, with the most common tumor being the insulin-producing insulinoma (Halperin et al., 2015). The autonomous production of excessive amounts of insulin, which results in hypoglycemia, is the classical feature of this tumor, and β-cell adenomas cannot decrease insulin secretion in the presence of hypoglycemia. The most critical diagnostic criterion is the detection of an inappropriately elevated plasma insulin level under conditions of hypoglycemia. The diagnosis of insulinoma therefore requires confirmation of the presence of hypoglycemia with evidence of inappropriate insulin secretion and the identification of a pancreatic mass by medical imaging or angiography (Vinik, 2014).

Delays to a diagnosis can be caused by a number of factors (Wexler et al., 2018). For example, insulinoma can exhibit various neurogenic and neuroglycopenic symptoms. These symptoms also mimic neuropsychiatric symptoms, which include unconsciousness, confusion, seizures, personality change, and bizarre behavior in most patients (Cryer, 2005). In addition, over half of the patients with these symptoms are initially misdiagnosed with neuropsychiatric disorders, such as epilepsy. Presentation is usually insidious with neuroglycopenia and fasting hypoglycemia. Normal insulin levels therefore do not rule out the disease because absolute insulin levels are not elevated in insulinoma patients. As this study revealed, this might lead to a delay in diagnosis. Diagnostic delays are therefore related to the fact that the symptoms are similar to many common neurological and psychiatric disorders. A study of 1,067 insulinoma patients showed that most patients developed neuropsychiatric symptoms, including loss of consciousness, unresponsiveness, delirium, deep coma, dizziness, visual disturbances, coma, and epilepsy. In addition, insulinomas secrete insulin, causing temporary fluctuations in blood sugar levels. Blood glucose levels in patients with insulinoma can therefore sometimes appear to be normal (Sotoudehmanesh et al., 2007). Symptoms of hypoglycemia include neuroglycopenia (confusion, lethargy, bizarre behavior palpitations, personality change, decreased motor activity, transient neurological deficit, and gradual decline in cognition) and autonomic symptoms (sweating, tremor, palpitations, anxiety, weakness, and visual disturbance). Presentation is usually insidious with neuroglycopenia and fasting hypoglycemia. As with this study, this may lead to a delay in diagnosis as other neuropsychiatric diagnoses are first considered. In a retrospective study of 59 patients with histologically confirmed islet cell adenomas, the interval between the onset of symptoms and diagnosis ranged from 1 month to 30 years with a median of 24 months. A significant proportion (39%) of the patients was originally diagnosed with a seizure disorder. Furthermore, all the patients had symptoms of neuroglycopenia, and three quarters of them reported a relief of symptoms with food ingestion (Harrington et al., 1983). Despite these findings, rare cases of insulinoma presenting with seizures have been reported in previous studies (Crimmins et al., 1990; Park and Kim, 2014). Therefore, neuroglycopenia should be considered in all patients with refractory seizures. In our case, due to lack of typical symptoms of hypoglycemia, such as sweating and a feeling of hunger, the patient was diagnosed with fasting blood glucose and glycosylated hemoglobin at the time of admission, and this led to misdiagnosis as a complex partial seizure. Furthermore, during the diagnosis process, the CT scan special imaging of the patient’s pancreas lesions almost led to a missed diagnosis of the tumor.

The patient’s clinical manifestations, hypoglycemia, insulin/blood glucose > 0.4, pancreatic enhancement CT, and upper abdominal enhanced MRI results supported the diagnosis of an islet cell tumor. Blood sugar levels returned to normal after surgical removal of the tumor, and the symptoms completely disappeared. During the 1 year of follow-up, the patient did not receive any treatment, and there were no symptoms or attacks.

In conclusion, the patient experienced episodes of hypoglycemic seizures induced by a pancreatic insulinoma. This highlights the need for careful reassessment of all atypical and refractory seizures.

## Data Availability Statement

The datasets generated for this study are available on request to the corresponding author.

## Ethics Statement

Ethical review and approval was not required for the study on human participants in accordance with the local legislation and institutional requirements. The patients/participants provided their written informed consent to participate in this study. Written informed consent was obtained from the individual(s) for the publication of any potentially identifiable images or data included in this manuscript.

## Author Contributions

ZQ and DL collected the case and wrote the manuscript. JM, YH, and PX acquised and analyzed the imaging data. AZ reviewed and approved the final manuscript.

## Conflict of Interest

The authors declare that the research was conducted in the absence of any commercial or financial relationships that could be construed as a potential conflict of interest.
